# Diversification of *Vibrio anguillarum* Driven by the Bacteriophage CHOED

**DOI:** 10.3389/fmicb.2019.01396

**Published:** 2019-06-20

**Authors:** Marcela León, Constantina Kokkari, Katherine García, Daniel Castillo, Pantelis Katharios, Roberto Bastías

**Affiliations:** ^1^Laboratorio de Microbiología, Instituto de Biología, Pontificia Universidad Católica de Valparaíso, Valparaíso, Chile; ^2^Institute of Marine Biology, Biotechnology and Aquaculture, Hellenic Centre for Marine Research, Heraklion, Greece; ^3^Instituto de Ciencias Biomédicas, Facultad de Ciencias de la Salud, Universidad Autónoma de Chile, San Miguel, Chile; ^4^Marine Biological Section, University of Copenhagen, Helsingør, Denmark

**Keywords:** diversification, bacteriophage, bacteriophage resistance, *Vibrio anguillarum*, bacterial evolution, virulence

## Abstract

Bacteriophages are an important factor in bacterial evolution. Some reports suggest that lytic bacteriophages can select for resistant mutant strains with reduced virulence. The present study explores the role of the CHOED bacteriophage in the diversification and virulence of its host *Vibrio anguillarum*. Nine phage-resistant strains were analyzed for their phenotype and different virulence factors, showing alterations in their fitness, motility, biofilm formation, lipopolysaccharide profiles and/or protease activity. Seven of the nine phage-resistant strains showed virulence reduction in a *Sparus aurata* larvae model. However, this is not generalized since two of the resistant strains show equal virulence compared with the parental strain. The genomic analysis of representative resistant strains displayed that the majority of the mutations are specific for each isolate, affecting genes related to lipopolysaccharide biosynthesis, quorum sensing, motility, toxin and membrane transport. The observed mutations were coherent with the phenotypic and virulence differences observed. These results suggest that the CHOED phage acts as a selective pressure on *V. anguillarum*, allowing proliferation of resistant strains with different genotypes, phenotypes and degrees of virulence, contributing to bacterial diversification.

## Introduction

*Vibrio anguillarum* is a marine bacterium that causes vibriosis in more than 50 fish species worldwide, including some species of high economic importance. This bacterium uses several virulence factors to develop the infection, such as flagellum, hemolysins, proteases, iron-uptake systems and outer membrane proteins (OMPs) that participate in osmoregulation between the marine and host environments (Frans et al., 2011). As in other bacterial species, the ecology and evolution of *V. anguillarum* can be associated with the presence of their bacteriophages.

Bacteriophages or phages are viruses that infect bacteria and are estimated to be the most abundant biological entities on the planet (Fuhrman, 1999). Phages play an important role in the regulation of bacterial populations due to their lytic effect on bacteria (Bouvier and del Giorgio, 2007; Shapiro et al., 2010). They can also directly affect bacterial evolution through the processes of transduction and lysogenic conversion (Canchaya et al., 2003). It has been shown that lytic phages can exert a selective pressure over their host, facilitating bacterial diversification (Brockhurst et al., 2005; Middelboe et al., 2009) and even reducing virulence of phage-resistant mutants (León and Bastías, 2015).

To date, several phages that infect *V. anguillarum* have been isolated from different geographic locations (Higuera et al., 2013; Silva et al., 2014; Tan et al., 2014; Kalatzis et al., 2017). Phages can differentially influence biofilm formation in *V. anguillarum* (Tan et al., 2015) and genomic analyses suggest that prophages can influence the virulence of this species (Castillo et al., 2017, 2018a). Moreover, phages have been successfully used to control infections caused by *V. anguillarum* in different animal models (Higuera et al., 2013; Silva et al., 2014; Rørbo et al., 2018).

Higuera et al. (2013) isolated and characterized the first bacteriophage for this bacterium, designated CHOED, which was proposed as a potential antimicrobial agent to combat the vibriosis caused by *V. anguillarum*. In that study, the authors reported the proliferation of strains resistant to this lytic phage, raising the question whether lytic phages, and in particular CHOED, have a role in the virulence and diversification of *V. anguillarum*. The present study is an effort to address that question, showing that CHOED may act as a selective pressure on *V. anguillarum*, allowing proliferation of resistant bacteria with different genotypes, phenotypes and degrees of virulence and in consequence favoring the diversification of the species. The study advances the understanding of how bacteriophages can impact bacterial diversification and virulence.

## Materials and Methods

### Bacteria Strain, Bacteriophage and Culture Media

The *V. anguillarum* PF4 strain (serotype O3) used in this study was originally isolated from an aquaculture center in southern Chile (Silva-Rubio et al., 2008) and has been deposited in the Polish Collection of Microorganisms (PMC) under the accession code B/00045. The *Pseudomonas aeruginosa* strain, PAO1 (Stover et al., 2000), used in the biofilm formation assay was kindly donated by Dr. Jorge Olivares of the Institute of Biology at the *Pontificia Universidad Católica de Valparaíso*. The bacteria were grown in synthetic seawater (23.4 g/L NaCl, 24.7 g/L MgSO_4_ × 7H_2_O, 1.5 g/L KCl and 1.43 g/L CaCl_2_ × 2H_2_O) supplemented with 1% Bactotryptone (Gifco) and 0.5% yeast extract, unless otherwise indicated. This medium was supplemented with 1.5% agar when using solid culture medium.

The CHOED bacteriophage that naturally infects the *V. anguillarum* PF4 strain is a lytic phage belonging to the *Podoviridae* family and was isolated from molluscs in southern Chile (Higuera et al., 2013). Its genome has been sequenced previously (Romero et al., 2014).

### Isolation of *V. anguillarum* Strains Resistant to the CHOED Bacteriophage

A culture of the *V. anguillarum* PF4 strain in the early exponential stage (O.D._600__nm_ 0.05–0.1) was infected with the CHOED bacteriophage at a multiplicity of infection (MOI) over 100. The culture was then incubated for 10 min at room temperature. Then 100 μL of the infected culture was spread in plates previously inoculated with 100 μL of the phage in order to keep the potentially resistant strains in permanent contact with the phages. The plates were incubated for 48 h at 25°C and then three bacteriophage resistant colonies were selected and cultured consecutively three times in solid medium in the absence of phage. Experiments were repeated in three independent assays, so nine resistant mutants were selected in total.

The protocol was repeated for nine isolated *V. anguillarum* clones or natural variants, but the bacteriophage was replaced by saline serum (0.85% NaCl).

The frequency of bacterial resistance to the CHOED phage was obtained by calculating the quotient between the concentration of the PF4 strain grown in presence and absence of the phage, as has been reported previously (Higuera et al., 2013).

The bacteriophage resistance of the selected phage-resistant strains and bacteriophage susceptibility of natural variant clones was confirmed using a spot test on double agar plates (Kutter, 2009).

In order to confirm that the bacterial isolates were stable in their resistance through generations, the strains were passed through 10 consecutive subcultures in a liquid medium. Samples were then taken from each subculture to evaluate bacterial resistance against phage infection using the double agar method.

### Growth Curve and Biochemical Profile

In order to evaluate the growth of the nine CHOED-resistant bacteria and the nine natural variants, these and the parent strain were cultured in parallel in 200 μL at 25°C using 96-well polystyrene plates (TrueLine). The O.D._600__nm_ of the cultures was measured at 30 min intervals using a plate reader (Infinite M200 PRO, TECAN). The cultures were grown in quadruplicate. In order to identify potential differences in the biochemical profiles of the nine resistant strains compared to the parent strain, the BIOLOG GEN III MICROPLATE^TM^ test was used in accordance with manufacturer’s instructions.

### Evaluation of Virulence Factors

#### Motility

The bacterial motility was evaluated as described by O’Toole et al. (1996). Briefly, TSB plates (Merck) supplemented with 0.4% agar (Bacto^TM^) and 2.3% NaCl (Merck) were inoculated in the center with the corresponding cultures (O.D._600__nm_ ∼ 0.1). The motility halos were measured after 48 h of incubation at 25°C. These experiments were carried out in triplicate.

#### Proteolytic Activity

The proteolytic activity levels of the phage-resistant strains and the parent strain were evaluated by applying the method used by Norqvist et al. (1990) with some modifications. 1% gelatine plates (HIMEDIA) were inoculated with 2 μL of early exponential phase cultures (O.D. _600__nm_ ∼ 0.05) and incubated at 25°C for 48 h, the dishes were treated with Frazier reagent (12.5% of HgCl_2_ in 1 M HCl) covering the entire surface. The diameter of the transparent halo around the bacterial colony indicating proteolytic activity was then measured and the results were compared with those obtained for the parent strain. These experiments were carried out in triplicate.

#### LPS Profiles

The LPS profiles were analyzed following the method of Hitchcock and Brown (1983) with few modifications. After centrifugation (8,000 rpm for 10 min at 4°C) the bacterial pellets were resuspended in PBS buffer until an O.D._600__nm_ ∼ 1.0. Then, 1.2 mL of each suspension were taken and centrifuged at 8,000 rpm for 10 min, discarding the supernatant and storing the pellet at –20°C overnight. Then each pellet was resuspended in 150 μL of lysis buffer (4% beta mercaptoethanol, 2% SDS, 10% glycerol and 0.002% bromophenol blue in 1 M Tris–HCl, pH 6.8). The samples were then boiled for 10 min and centrifuged at room temperature at 13,000 rpm for 15 min. 10 μL of each supernatant was taken and diluted in 90 μL lysis buffer without beta mercaptoethanol or SDS. 12 μL of proteinase K (20 mg/mL) were added to each sample and these were incubated for 1 h at 60°C. Finally, the LPS profile was visualized using SDS-PAGE electrophoresis gel stained with silver nitrate.

#### Biofilm Production

Biofilm formation was analyzed using the protocol described by O’Toole (2011) with modifications. Briefly, bacterial cultures of O.D._600__nm_ ∼ 0.6 were diluted 100 times and 100 μL of each culture were inoculated in 96-well plates. After incubation at 25°C for 24 h, the liquid from each well was discarded. Each well was washed thoroughly with distilled water and 125 μL of 1% crystal violet solution (Merck) was added. The plate was incubated at room temperature for 15 min and then washed and left to dry overnight. Subsequently, 125 μL of 30% acetic acid (Merck) was added to each well and left to rest at room temperature for 15 min. Finally, the contents of the plate were transferred to a new plate and the O.D._550__nm_ was measured using a plate reader (Infinite M200 PRO, TECAN). The PAO1 strain of *P. aeruginosa* was used as a positive control for the assay. These experiments were carried out in quadruplicate.

#### Hemolytic Activity

In order to analyze the hemolytic activity of the bacteria, a fresh culture of each strain was adjusted to ∼ 0.1 O.D._600__nm_ with saline solution (0.85% NaCl) and used to inoculate fish-blood agar plates (5% blood obtained aseptically from healthy seabass broodstock fish and used fresh in Oxoid blood agar base). The plates were then incubated at 25°C for 48 h. Hemolytic activity was quantified by the diameter of the hemolytic halo (Rowe and Welch, 1994).

#### Estimates of Siderophore Production

In order to evaluate siderophore production, the bacteria were cultured in a CM9 minimal medium without iron (0.6% Na_2_HPO_4_, 0.3% KH_2_PO_4_, 2.3% NaCl, 0.1% NH_4_Cl, 1 mM MgSO_4_, 0.1 mM CaCl_2_ and 1% glucose) until reaching a O.D._600__nm_ ∼ 0.3. Then, 1 mL of each culture was centrifuged at 8,000 rpm for 5 min and the supernatant was transferred to new test tube, mixed with 0.5 mL of CAS reagent (Pérez-Miranda et al., 2007) and incubated for 15 min. The strain was considered a siderophore producer if the medium changed from blue to yellow. To compare the level of siderophore production among the different strains, the O.D._630__nm_ was measured using a plate reader (Infinite M200 PRO, TECAN). The percentage of siderophore units for each strain was estimated as described by Bholay et al. (2012), using as reference the mixture of 0.5 mL of fresh CM9 medium without the 0.5 mL of CAS reagent.

#### OMP Profiles

Outer membrane protein extraction was performed according the protocol described by Busico-Salcedo and Owens (2013), with some modifications. Briefly, the bacteria were cultured overnight at 25°C with agitation stirring. The culture was centrifuged at 10,000 rpm for 15 min at 4°C and the supernatant discarded. The pellet was washed with buffer 1 (10 mM Tris–HCl, pH 8) and resuspended in 1 mL of the same buffer. The sample was then sonicated in ice using 6 pulses of 10 s interspaced with 20 s pauses between each pulse. The sample was then centrifuged at 7,000 rpm for 5 min at 4°C. The supernatant was recovered, taking care not to touch the pellet and it was then centrifuged again at 13,000 rpm for 45 min at 4°C. The second supernatant was discarded and the pellet was resuspended in 500 μL of buffer 2 (10 mL Tris–HCl at pH 8, MgCl_2_ 10 mM and 2% (v/v) Triton X-100). The mixture was incubated for 30 min at 37°C with occasional stirring. Finally, the sample was centrifuged again at 13,000 rpm for 45 min at 4°C, the supernatant was discarded and the pellet was resuspended in 30 μL of buffer 3 (100 mM Tris–HCl at pH 8 and 2% SDS). The samples were stored at –20°C and the OMP profiles were visualized by SDS-PAGE and Coomassie staining.

#### Electron Microscopy

An analysis was conducted, through transmission electron microscopy, to detect potential alterations in the flagellum in the different mutants resistant to the CHOED phage. Resistant mutants and the PF4 strain were grown for 24 h at 25°C on plates with TSB (MerK) supplemented with 1.5% agar and 2.3% NaCl. Each of the strains was transferred in parallel to an eppendorff containing 250 μL of sterile distilled water and the suspension was homogenized manually. Then 15 μL of each strain was taken and fixed in copper racks for 30 s. Subsequently, they were dried at 37°C for 15 min and finally, the bacteria were observed using a transmission electron microscope (Tecnai 12 Biotwin, Philips) and negative staining (McGee et al., 1996).

### Gilthead Seabream Larvae (*Sparus aurata*) Experiments

The virulence of the phage-resistant strains and the natural variants of parental strain were evaluated taking into consideration the 3Rs: Replace, Reduce and Refine. All experiments were performed in accordance with the National legislation concerning laboratory animals in registered facilities for experimentation. The Hellenic Centre for Marine Research (HCMR) has registered facilities for fish experiments (EL91-BIO-exp04). Briefly, recently fertilized gilthead seabream eggs of the same batch were collected from the broodstock facility of HCMR and washed twice with sterile seawater. The eggs were transferred aseptically to 96-well plates along with 200 μL of sterile seawater, placing them individually in the wells as described by Panini et al. (2001) using only the 60 central wells of each plate. The remaining wells around the edge were filled with 200 μL of sterile seawater without eggs. The eggs were then challenged with the different bacterial strains at a final concentration of approximately 5 × 10^6^ CFU/mL resuspended in sterile seawater. The challenge was conducted in darkness at 20°C and mortality of the larvae was monitored daily for 7 days. Three plates were analyzed per bacterial strain, with a total of 180 eggs per case. Once the experiment had been completed, 3 samples were collected from each plate to determine the load of vibrios using TCBS agar (Difco^TM^).

### DNA Extraction and Sequencing

Bacterial DNA from wild-type and phage-resistant strains PF4-R4, PF4-R6 and PF4-R8 was extracted using Kit Wizard^®^ (Promega) according to the manufacturer’s protocol. The complete genomes of *V. anguillarum* strains, were sequenced using IlluminaHiSeq platform (Macrogen, Corea) with paired-end read sizes of 101 bp. Library construction, sequencing and data pipelining were performed in accordance with the manufacturer’s protocols.

### Computational Analyses

We sequenced the genomes of 4 bacterial strains: The parent PF4-WT strain and 3 phage-resistant isolates (PF4-R4, PF4-R6 and PF4-R8). Sequences were trimmed to remove both Illumina specific adapters and bad quality bases in single reads using Geneious software (version 9.1.8) (Kearse et al., 2012).

The Illumina data was assembled using both, reference-based and *de novo* method. For the reference-based method, reads of the parent strain were mapped using the chromosomes I and II of *V. anguillarum* PF4 strain as reference genome (GenBank accession number CP010080 [chromosome I] and CP010081 [chromosome II], February 2017) with Geneious (version 9.1.8) (Kearse et al., 2012). The resulting sequence was then used as a reference for alignment to the phage-resistant strains. For the *de novo* method, reads of the parental and mutant strains were assembled using CLC Genomics Workbench version 10 (Bio CLC). Genome comparison of PF4-WT with the phage-resistant isolates was done using MAUVE v2.3.1 software (Darling et al., 2004). Only mutations present in at least 80% of the reads and present in the genome of the strains assembled by both methods, were considered for further analysis.

Annotation of the genomes was done with the NCBI Prokaryotic Genome Automatic Annotation Pipeline (PGAAP) (Tatusova et al., 2016). Alternatively, genomic annotation was done by rapid annotation using subsystem technology (RAST) (Overbeek et al., 2014).

Prediction of the subcellular localization of bacterial mutant proteins was achieved using PSort v3.0b (Yu et al., 2010). An analysis for the presence of transmembrane helices (TMH) was performed by TMHMM v2.0c (Krogh et al., 2001) and predictions of signal peptides were obtained using SignalIP v3.0 (Emanuelsson et al., 2007). Finally, prophage-related sequences were identified by running bacterial genomes in PHASTER (Arndt et al., 2016).

### Statistical Analysis

Statistical significance of the data obtained from the tests of motility, proteolytic activity and the production of exopolysaccharide (EPS) for biofilm formation was determined using one-way ANOVA analysis followed by the Dunnett multiple comparison test with a level of significance of *p* = 0.05 (using the program Graph Pad 6). The statistical significance of the data obtained from the challenges of fish larvae was analyzed using Chi-squared analysis (χ^2^) with a level of significance of α = 0.05^1^ from the cumulative mortalities at the end of the experiment (Bergh et al., 1997).

To compare the variation of accumulated mortality data, obtained in the challenges conducted with the phage-resistant strains and those obtained in the challenges performed using natural variants of *V. anguillarum*, the coefficient of variation (CV) was calculated by dividing the standard deviation of the mortalities by the average of the mortalities obtained in each case. This result was multiplied by 100 (Reed et al., 2002).

### Accession Number

The genomic sequences of all strains were deposited in the NCBI GenBank. Accession numbers for chromosomes are listed in Supplementary Table S8.

## Results

### Selection, Growth and Biochemical Profile of *V. anguillarum* Strains Resistant to the CHOED Bacteriophage

Mutant strains of *V. anguillarum* that were resistant to infection by the CHOED bacteriophage were selected to evaluate potential changes in their phenotype and virulence. The bacteriophage-resistant mutants appeared with a frequency of 8.19 × 10^-6^. Nine phage-resistant mutants were isolated for further analysis and named PF4-R1 to PF4-R9 after the parental PF4 strain. The resistant mutants retained resistance to CHOED infection through 10 consecutive subcultures and no morphological changes were observed in their colonies.

**FIGURE 1:**
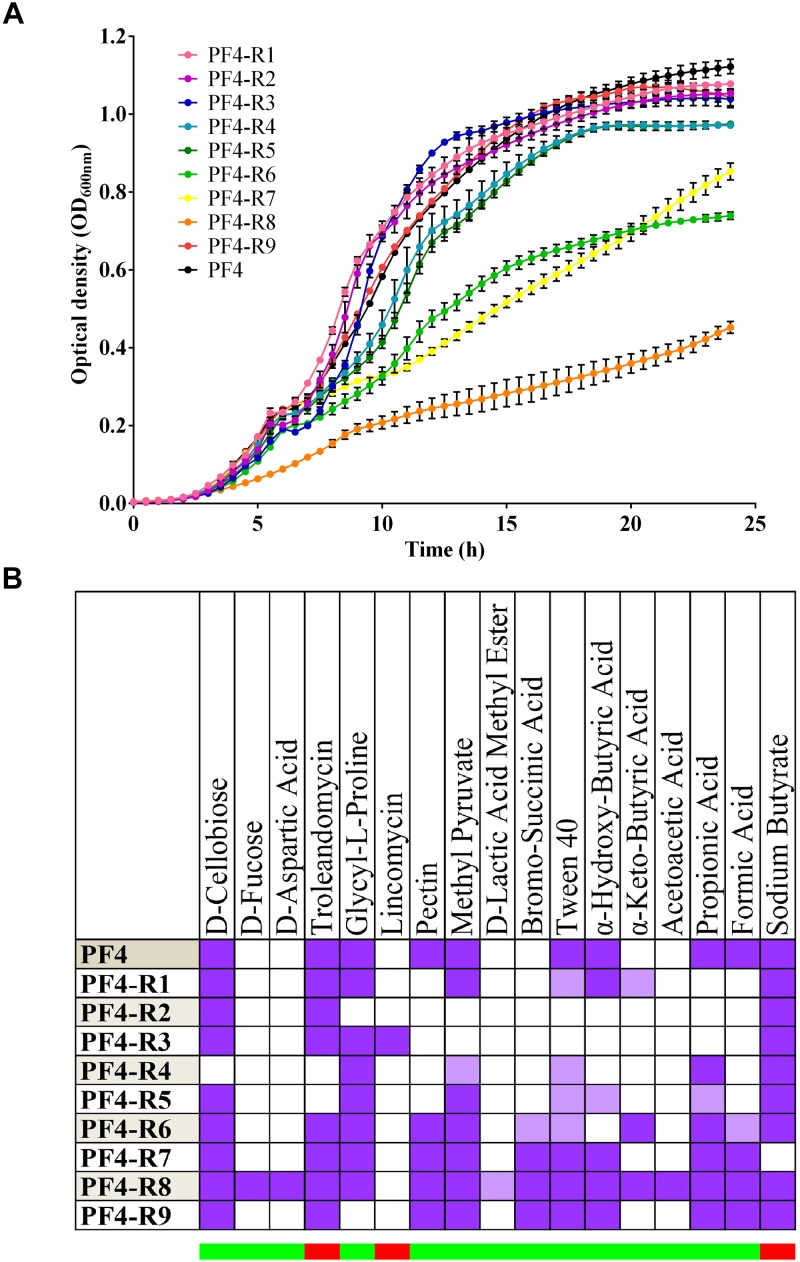
Phenotypic diversity of *V. anguillarum* strains resistant to the CHOED phage. **(A)** Growth curve of bacteriophage-resistant *V. anguillarum* strains. PF4 is the *wt* parental strain. The error bars indicate standard deviation. The cultures were performed in quadruplicate. **(B)** Differences in biochemical profile of bacteriophage-resistant *V. anguillarum* strains. Differences observed in biochemical profile among the different strains using BIOLOG GEN III MICROPLATE^TM^. PF4 is the *wt* parental strain. The upper part shows the different chemical compounds used in the corresponding test. The purple color indicates a positive reaction (growth), white indicates a negative reaction (no growth) and light purple indicates an intermediate reaction. The lower bar indicates whether it is a test for susceptibility to the compound (red) or for its use as a source of carbon (green).

The resistant mutants PF4-R6, PF4-R7 and PF4-R8 exhibited growth impairment, with the latter most affected, reaching less than 50% of the O.D._600__nm_ obtained by parental PF4 strain after 24 h of incubation (Figure 1A). Strains PF4-R4 and PF4-R5 showed a slight decrease in growth compared to PF4, while the remaining strains showed no apparent alterations. These results show that the strains resistant to CHOED had a diverse range of growth.

In addition, the BIOLOG GEN III system was used to evaluate the biochemical profile of the different mutant strains resistant to CHOED. As observed with the bacterial growth, this second analysis showed that the resistant mutants had a diverse range of biochemical profiles, although the majority of the tests included in the BIOLOG assay were not altered compared to the parent strain (Figure 1B). 17 out of 94 tests used in the analysis showed differences in at least one of the resistant mutants (from positive to negative/borderline reaction, or from negative to positive/borderline reaction), representing 18% of all tests. Since all the resistant mutants showed a different biochemical profile, it was not possible to correlate a specific profile with resistance to the phage. Among the changes observed in the biochemical profiles, 14 were related to the capacity to use a particular compound as a carbon source, while the rest correspond to susceptibility tests. In the majority of the cases the alterations corresponded to the loss of the ability to use a specific carbon source (24 times among the nine phage-resistant strains), where the ability to grow in pectin or formic acid were the most common loss. These results show that the diversity observed in the bacterial growth is also reflected in their ability to use different carbon sources and susceptibility to environmental conditions or compounds. However, since there are phage-resistant strains that do not show alteration in their growth, the resistance to the CHOED bacteriophage is not necessarily related to a lower fitness in the resistant strains.

### Virulence Factors in CHOED-Resistant Mutants of *V. anguillarum*

**FIGURE 2:**
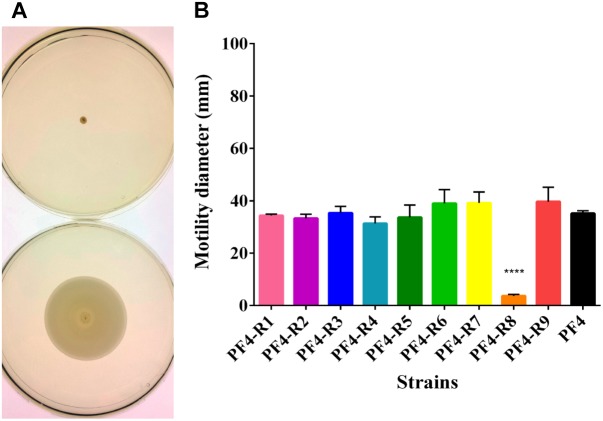
Motility of *V. anguillarum* strains resistant to the CHOED phage. **(A)** Motility of resistant PF4-R8 strain (upper) and parental PF4 strain (lower). **(B)** Motility halo for each of the resistant strains. Tests were carried out in TSB medium supplemented with 0.4% agar and 2.3% NaCl. Cultures were incubated for 48 h at 25°C. Error bars represent standard deviation of triplicates. The results of the strain marked (^∗∗∗∗^) are significantly different from the parent strain (*p* < 0.0001).

Several virulence factors, such as motility, LPS, extracellular proteases and hemolysin production have been described to play an important role in the infection by *V. anguillarum* (Frans et al., 2011; Naka and Crosa, 2011). In the analysis of motility, the only strain of the resistant mutants that showed a significant reduction was PF4-R8, with an 89.5% reduction in displacement compared to PF4 (Figure 2).

**FIGURE 3:**
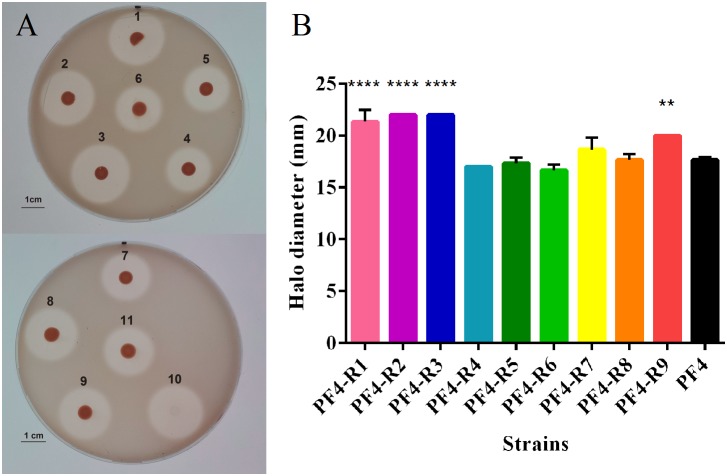
Protease production of *V. anguillarum* strains resistant to the CHOED phage. **(A)** Protease production of different mutant strains resistant to CHOED. 1: PF4-R1; 2: PF4-R2; 3: PF4-R3; 4: PF4-R4; 5: PF4-R5; 6: parental PF4 strain; 7: PF4-R6; 8: PF4-R7; 9: PF4-R8; 10: PF4-R9 (colony removed by buffer addition); 11: parental PF4 strain. **(B)** Diameter of the proteolytic activity halo for the different resistant strains. Error bars represent standard deviation of triplicates. (^∗∗^) Significantly different from the parental PF4 strain (*p* < 0.01); (^∗∗∗∗^) Significantly different from the parental strain (*p* < 0.0001).

Another important factor of virulence evaluated was the presence of extracellular proteases. The analysis showed that PF4-R1, PF4-R2, PF4-R3 and PF4-R9 had significant increase in extracellular protease production, 21, 25, 25, and 13%, respectively in comparison with the parental PF4 strain, according to the degradation halo observed on gelatine plates (Figure 3).

**FIGURE 4:**
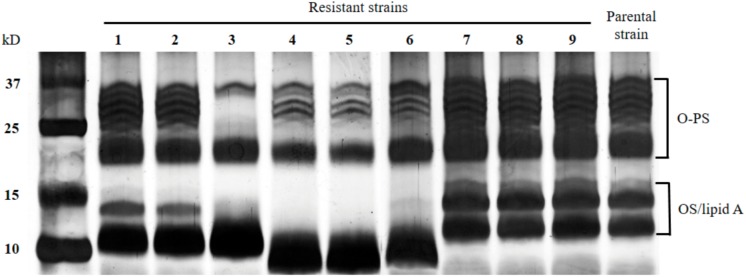
LPS profile. The LPS components of the mutants resistant to CHOED phage and the parental PF4 strain were separated by 18% SDS-PAGE and stained with silver nitrate. The molecular weight marker KaleidoscopeTM (BIORAD) was included in the first left lane, whose sizes are shown in the figure. (1) PF4-R1, (2) PF4-R2, (3) PF4-R3, (4) PF4-R4, (5) PF4-R5, (6) PF4-R6, (7) PF4-R7, (8) PF4-R8, (9) PF4-R9 and (10) parent PF4 strain.

Analysis of the Lipopolysaccharide (LPS) profiles of the resistant mutants showed that the parental PF4 strain presented a group of upper bands of high molecular weight that represent the O-PS and a group of lower bands of low molecular weight that represent the core OS/lipid A portion (Stroeher et al., 1998). The resistant mutants PF4-R7, PF4-R8 and PF-R9 showed no alterations compared to PF4 strains. However, PF4-R1 and PF4-R2 strains showed alterations in bands of the core OS/lipid A portion. While the PF4-R3 strain displayed alterations in both O-PS and OS/lipid A portions. The resistant mutants PF4-R4, PF4-R5 and PF4-R6 presented only slight alterations in the O-PS portion, but marked differences in the core OS/Lipid A section with lower molecular bands, suggesting an impairment in this portion of the LPS (Figure 4).

**FIGURE 5:**
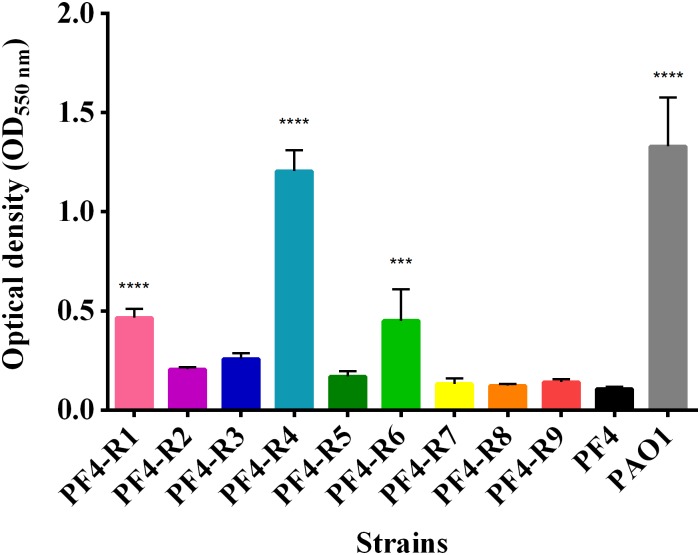
Biofilm formation of *V. anguillarum* strains resistant to the CHOED phage. The analyses of each strain carried out in quadruplicate in 96-well plates staining the exopolysaccharide in the biofilms with 0.1% crystal violet as described in materials and methods. Error bars represent standard deviation. (^∗∗∗^) Significantly different from parental PF4 strain (*p* < 0.001); (^∗∗∗∗^) significantly different from the parental strain PF4 (*p* < 0.0001).

Similarly to other observed virulence factors, the bacteriophage-resistant mutants showed different levels of biofilm formation. PF4-R1, PF4-R4 and PF4-R6 strains showed a significant increase in biofilm formation compared to PF4 (Figure 5). In the case of PF4-R4, this increase was 11-fold higher than the parental strain, which is equal to the biofilm produced by *P. aeruginosa*, a bacterium widely recognized as a biofilm producer (Ciofu et al., 2012).

Unlike the previous analyses, no significant differences were observed in hemolysin production, siderophore production, or the OMP profiles of the resistant mutants compared to parental strain (see Supplementary Figures S1A,B, S2, respectively). The sum of all these results shows that the CHOED-resistant mutants of *V. anguillarum* had different phenotypes with different altered virulence phenotypes, though the analysis of these variations did not reveal a clear pattern that could be related to bacteriophage resistance. An alteration in virulence factors does not necessarily imply alteration in the virulence of the bacteria. Therefore, the virulence of the different bacteriophage-resistant mutants was evaluated in a fish larvae model.

### *Vibrio anguillarum* Virulence of CHOED-Resistant Mutants

**FIGURE 6:**
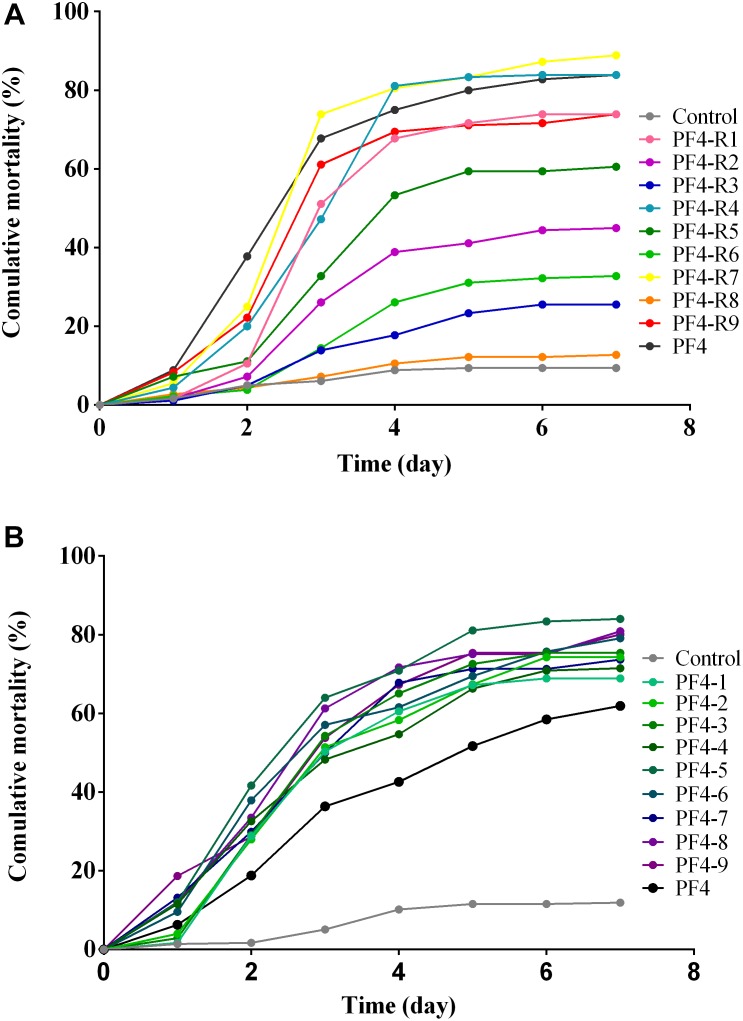
Challenge in Gilt-Head Sea bream (*S. aurata*) larvae with *V. anguillarum* strains. **(A)** Strains resistant to the CHOED phage. **(B)** Clones of parental strains. Test carried out in 96-well plates using one ova/larva per well. Mortality of the larvae was monitored daily for 7 days. All strains inoculated to attain the same final concentration (∼5 × 10^6^ CFU/mL). The control corresponds to larvae with the same volume of sterile seawater.

In order to determine potential changes in the virulence of the bacteriophage-resistant mutants, larvae of gilthead seabream (*S. aurata*) were exposed to the different resistant mutants. The results obtained from these experiments reveal that, seven of the nine mutants showed a decrease in their virulence in comparison to the parental PF4 strain. However, similarly to some of the previous analyses, there is a wide variety in the degree of virulence among the different resistant mutants (Figure 6A). The most notable is the mutant PF4-R8, which was totally avirulent and showed no significant differences from the control performed without the bacteria (*p* > 0.05). The mutants PF4-R4 and PF4-R7, on the other hand, were fully virulent and showed no significant differences from the parental PF4 strain at the end of the experiment (*p* > 0.05). The other mutant strains resistant to the phage showed intermediate degrees of virulence that were significantly different from both PF4 and the control without bacteria (*p* < 0.05) (see Supplementary Table S1). These results suggest that the resistant strains showed a significant decrease in their virulence. However, this decrease in virulence was not a generalized fact, given that two of the phage-resistant strains retained the virulence of the parental strain.

To verify that the variation in virulence observed in the strains of *V. anguillarum* mutants was a result of the selection pressure exerted by the CHOED phage and not due to natural variation within this species, different *V. anguillarum* clones were isolated under the same conditions with that of the resistant mutants, but without the presence of the CHOED bacteriophage. These natural variants of *V. anguillarum* were used to challenge gilthead seabream (*S. aurata*) larvae. The results showed that although there is a degree of variation, the data of the accumulated mortality revealed no significant differences between the different natural variants, showing a lower variability in their virulence than the resistant mutants (Figure 6B and Supplementary Table S2). Furthermore, when comparing the variation of accumulated mortality data by the coefficient of variation (CV), natural variants (8.7%) had a CV 5 times lower than that obtained with phage resistant strains (47%) (see Supplementary Table S3). This lower variability among the natural variants of *V. anguillarum* was also observed in other characteristics such as bacterial growth, biofilm formation and motility, where the coefficient of variation of natural variants of *V. anguillarum* was much lower than the CV of the resistant mutants (see Supplementary Figure S3 and Supplementary Tables S4, S5). Taken together, these results strongly suggest that the diversity in phenotypes and virulence observed among the resistant mutants of *V. anguillarum* are associated with the resistance to the CHOED phage.

### Identification of Mutations in Phage-Resistant *Vibrio anguillarum* Strains

In order to link the different phenotypes and degrees of virulence observed among the resistant mutants, the PF4-R4, PF4-R6 and PF4-R8 strains (virulent, mildly virulent and avirulent, respectively) were selected for sequencing and genome analysis together with the parental strain PF4. The genomic analysis revealed 39 independent mutations in the sequenced strains, of which 72% of mutations were in chromosome I and 28% in chromosome II. Of the total mutations observed, 31% were point mutations and 69% were indels [deletions (38%) or insertions (31%)] within a range of 1–9592 bp, corresponding to 12 were point mutations, 25 small indels (between 1 and 67 bp) and 2 large indels (8,905 and 9,582 bp), respectively. The majority the point mutations caused amino acid changes (AC) (75%) and only 3 point mutations created stop codon (SC) (25%). On the other hand, 14% of the small indels caused amino acid insertion (AI), 48% caused reading frame shifts (FS) and 38% caused reading frame shifts with anticipated stop codons (^e^FS). The large deletions caused full gene deletions (D) (see Supplementary Table S6).

The resistant mutants PF4-R4, PF4-R6 and PF4-R8 contained 7, 22 and 10 mutations, respectively, corresponding to 18, 56 and 26% of the total mutations detected among the sequenced strains. The mutant PF4-R6 was the strain that accumulated the most mutations; with more than a half of the total mutations observed among the selected resistant strains (see Supplementary Table S6). In total, the different mutations observed affected genes coding for 48 proteins, with different functions such as biosynthesis of LPS, regulation of genetic expression, quorum sensing, metabolism, toxin, membrane transport and some uncharacterized hypothetical proteins.

**FIGURE 7:**
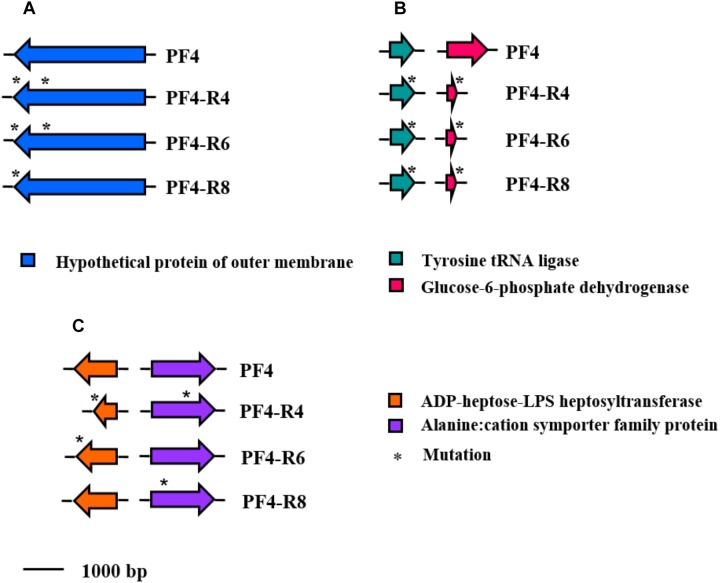
Schematic representation of the distribution of specific mutations in phage-resistant strains of *V. anguillarum*. Genes are represented as arrows and point mutations are represented as asterisks. Functions for genes are shown in colored boxes. **(A)** Mutation shared by all phage-resistant strains on the chromosome I (accession number WP_017049502.1); **(B)** Mutation shared by all phage-resistant strains on the chromosome II (accession numbers NP_230280.1 and NP_233281.1, respectively). **(C)** Mutation shared by two of the phage-resistant strains (accession numbers WP_019281202.1 and NP_797001.1, respectively).

The mutations were distributed differently in the resistant strains of *V. anguillarum* sequenced. However, all strains shared mutations in the same three genes: (I) A insertion of 2 bp in the gene coding for the glucose-6-phosphate dehydrogenase protein (NP_233281.1), (II) a deletion in the dehydrogenase Tyrosine tRNA ligase gene (NP_230280.1) and (III) a deletion in a hypothetical OMP (WP_017049502.1) (Figure 7). The analysis of the latter gene reveals that the hypothetical protein (1036 amino acids) would have an internal region (amino acids 1–266), a transmembrane region (amino acids 267–289) and an external region (amino acids 290–1036). The resistant mutants PF4-R4, PF4-R6 and PF4-R8 presented deletions of 6, 9 and 27 bp, respectively in the carboxyl terminal region corresponding to the external region of the protein, generating alterations in its length.

The resistant mutants PF4-R4 and PF4-R6 shared deletions in the ADP-heptose-LPS heptosyltransferase gene (WP_019281202.1) related to LPS biosynthesis. These mutations produced a frame shift with a premature stop codon in PF4-R4 and only a premature stop codon in PF4-R6. Similarly, the PF4-R4 and PF4-R8 strains showed point mutations in the Alanine:cation symporter family protein gene (NP_797001.1), causing amino acid change (Figure 7).

**FIGURE 8:**
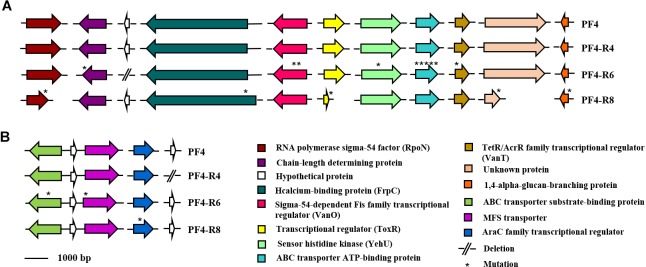
Schematic representation of the distribution of specific mutations in phage-resistant strains of *V. anguillarum*. Genes are represented as arrows, point mutations are represented as asterisks and deletion mutations are represented by short lines. Functions for genes are shown in colored boxes. **(A)** Mutations in chromosome I (accession numbers NP_232157.1, WP_017048278.1, WP_017049544.1, WP_013856939.1, WP_010320686.1, WP_013857308.1, WP_010319157.1, NP_798862.1, WP_017043694.1 and WP_017046665.1, respectively) **(B)** Mutations in Chromosome II (accession numbers WP_017048771.1, WP_013867897.1, WP_000931488.1, WP_017044929.1 and WP_001899004.1, respectively).

**FIGURE 9:**
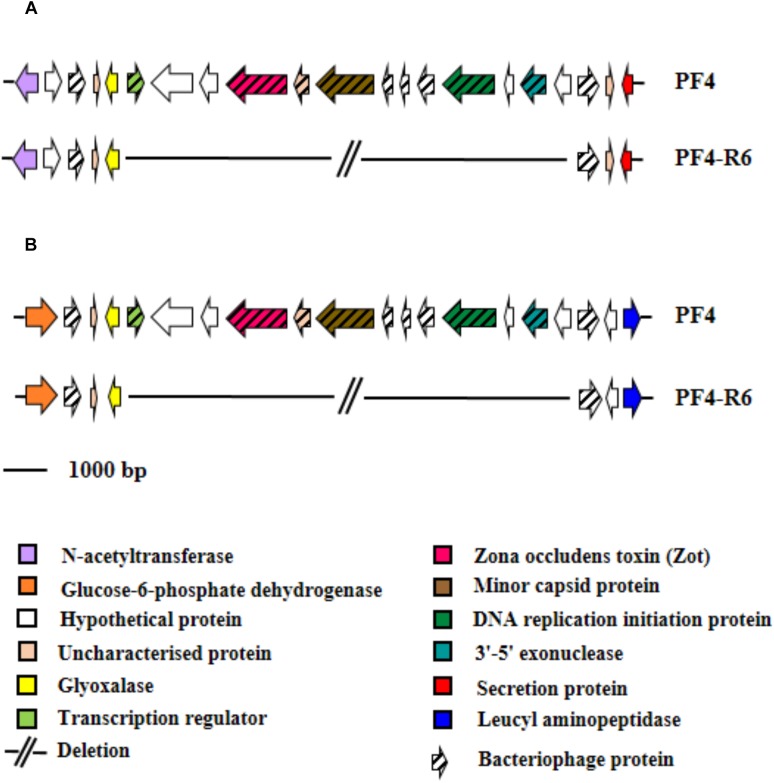
Schematic representation of the distribution of specific mutations in phage-resistant strains of *V. anguillarum*. Genes are represented as arrows, deletion mutations are represented by short lines, term of the chromosome is represented by perpendicular line and phage proteins are represented by arrow with thick line. Functions for genes are shown in colored boxes. Specific region that presented a large deletion in PF4-R6 strain. **(A)** Chromosome I (accession numbers). **(B)** Chromosome II (accession numbers WP_019820677.1, WP_006880472.1, WP_017041958.1, WP_019281837.1, WP_017049549.1, WP_017041960.1, WP_017049548.1, WP_017049547.1, WP_017049546.1, WP_017049545.1, WP_009706113.1 and WP_017049544.1, respectively).

The vast majority of mutations were specific and unique for each resistant mutant sequenced. For instance, the strain PF4-R8 showed a 67 bp deletion in the RNA polymerase sigma-54 factor gene (*rpoN*) (NP_232157.1), which caused a frameshift with a premature stop codon. The PF4-R6 strain showed several unique mutations such as a 7 bp deletion in the LPS chain-length determining protein gene (WP_017048278.1), causing a premature stop codon, a 5 bp insertion in the gene coding for the transcriptional regulator (VanT) (WP_017043694.1) and two point mutations in transcriptional regulator (VanO) (WP_010320686.1), which caused frameshift with a premature stop codon and amino acid changes, respectively. This strain also presented mutations in genes coding for proteins associated with transmembrane substrate transport such as ABC transporter ATP-binding proteins (NP_798862.1 and WP_017048771.1), ABC transporter substrate-binding protein and transporter MFS (major facilitator superfamily) (WP_000931488.1) (Figure 8). Finally, this mutant had two large deletions, one of 8,905 bp and another of 9,582 bp in chromosome I and II, respectively. The genome analysis with the PHASTER software showed that the deleted genes in the chromosome I and II of PF4-R6 correspond to a prophage-like element of 9.8 kb linked to a Zot-like toxin present in the parental PF4 strain (Figure 9). The PF4-R6 had lost most of the prophage genes in both chromosomes, including glyoxalase, transcription regulator, Zona occludens toxin, minor capsid protein, DNA replication initiation protein, 3′–5′ exonuclease and hypothetical proteins (Figure 9). This analysis also revealed that all sequenced strains carried a second intact prophage of 53 kb in chromosome II (see Supplementary Table S7).

## Discussion

The present study focused on an area that has recently become of general interest, i.e., how lytic bacteriophages can influence the evolution and virulence of their host bacteria by acting as a selective pressure (Middelboe et al., 2009; Hosseinidoust et al., 2013a,b; Avrani and Lindell, 2015). Our results provide evidence that in the case of *V. anguillarum*, the CHOED bacteriophage promotes diversification of the species by selecting for variants resistant to the phage with different phenotypes, genotypes and virulence.

### Diversity of Phenotypes in *V. anguillarum* Mutants Resistant to the CHOED Phage

The diversity among the different bacteriophage-resistant mutants can be seen in the different analyses performed, such as the growth curves and the biochemical profiles (Figure 1B). These results agree with several other reports that also showed alterations in growth (Capparelli et al., 2010a,b) and biochemical profiles (Middelboe et al., 2009; Christiansen et al., 2016) in phage-resistant bacteria. These phenotypic differences are also reflected in the different virulence factors described for *V. anguillarum* (Frans et al., 2011; Naka and Crosa, 2011; Castillo et al., 2017; Hickey and Lee, 2018). The resistant mutants presented differences in five out of the eight tests performed in this study. For instance, four out of the nine resistant mutants included in the study (PF4-R1, PF4-R2, PF4-R3 and PF4-R4) presented significantly higher extracellular protease production in comparison to the parental PF4 strain (Figure 3), which in *V. anguillarum* has been associated with mucus degradation and initial colonization of the host (Norqvist et al., 1990; Denkin and Nelson, 2004). These results agree with those obtained by Hosseinidoust et al. (2013b) that demonstrated that mutants of *P. aeruginosa* resistant to the phages PP7 and E79 have increased production of at least one of the tested virulence factors, such as protease, elastase, phospholipase C and hemolysins.

Lipopolysaccharide, a major component of the outer membrane of the Gram negative bacterial cell, consisting of lipid A, core oligosaccharide (OS), and O-polysaccharide (O-PS) (Raetz and Whitfield, 2002). In *V. anguillarum* LPS has been associated with complement evasion (Boesen et al., 1999; Welch and Crosa, 2005). Besides, several bacteriophages use LPS as bacterial receptor (Michel et al., 2010; Kiljunen et al., 2011). Here we found that the resistant mutants PF4-R1, PF4-R2, PF4-R3, PF4-R4, PF4-R5 and PF4-R6 presented alteration in the LPS, however, PF4-R7, PF4-R8 and PF4-R9 did not show differences compared with the parental PF4 strain (Figure 4). Therefore, is rather unlikely that the CHOED bacteriophage use LPS as receptor. Conversely, all three resistant mutants sequenced PF4-R4, PF4-R6 and PF4-R8 presented mutations in the same hypothetical OMP (WP_017049502.1), therefore this protein could be a good candidate as the receptor used by the CHOED bacteriophage to infect *V. anguillarum* (Figure 7), however, more experiments are need to confirm this hypothesis.

Biofilm formation is also considered a factor that favors virulence in many species of pathogenic bacteria (Hall-Stoodley et al., 2004). In the case of bacteria of the *Vibrio* genus, it is believed that the ability to form biofilms can help the bacteria persist in the environment and resist the action of antimicrobials and disinfectants. A recently published study looked at the effect of the phage ΦH20 on the BA35 *V. anguillarum* strain and the phage KVP40 on the PF430-3 *V. anguillarum* strain (Tan et al., 2015). These researchers found that the phages affect their host bacteria differently. While the bacteriophage ΦH20 inhibits biofilm formation in the host strain, KVP40 stimulates biofilm formation in the PF430-3 strain, which was suggested to be a defense mechanism of the bacterium. Our results showed that three of the resistant mutants (PF4-R1, PF4-R4 and PF4-R6) had increased biofilm production (Figure 5), suggesting a similar defense mechanism to PF430-3. However, in this case biofilm production may be explained by genetic modifications and not due to a physiological change as it is proposed for the PF430-3 strain. Moreover, as not all the resistant mutants included in this study presented alteration in biofilm production, as such it cannot be stated definitively as a common bacteriophage defense mechanism in *V. anguillarum* or specifically against CHOED.

In addition, the low phenotypic variability observed with the natural variants of *V. anguillarum* contrasted with the variability observed with the bacteriophage-resistant mutants, suggesting that this variability may be associated to the resistance to the CHOED phage (Figure 6B and Supplementary Table S2).

### Reduction in the Virulence of *V. anguillarum* Mutants Resistant to CHOED Phage

The analysis showed a wide range of phenotypes and altered virulence factors in the different mutants of *V. anguillarum* that were resistant to the CHOED phage. This diversity in the resistant mutants was also corroborated *in vivo* by the experiments in gilthead seabream (*S. aurata*) larvae. Fish larvae have been used previously for infection with *V. anguillarum* (D’Alvise et al., 2013; Frans et al., 2013) and a study was recently published that uses fish larvae for evaluating virulence differences between strains of *V. anguillarum* (Frans et al., 2013; Rønneseth et al., 2017). The results obtained in the challenges revealed diversity in the degree of virulence (Figure 6). In spite of this diversity, seven out of the nine mutants analyzed showed a significant decrease in their virulence compared to the parental PF4 strain, which corresponds to 78% of the strains analyzed. This result is in agreement with several reports suggesting that resistance to bacteriophages is associated with a decreased virulence in the resistant bacteria (Heierson et al., 1986; Park et al., 2000; Santander and Robeson, 2007; Capparelli et al., 2010a,b; Friman et al., 2011; Laanto et al., 2012). These observations corroborate the notion that resistance to bacteriophages comes with a fitness cost for the bacterial host. This may be explained also by the fact that bacteriophages frequently use surface structures in the bacteria as receptors for infection, which are also virulence factors. Hence, bacteria that acquire resistance by modifying these receptors may also exhibit an effect on their virulence (León and Bastías, 2015). However, in this work two of the resistant mutants remained equally virulent as the parental PF4 strain (Figure 6), suggesting that a decrease in the virulence of phage-resistant mutants is not a universal feature. It remains unclear if this is a special characteristic in *V. anguillarum* or whether a similar situation occurs in other bacteria-phage systems. It is possible that bacteriophage resistant mutants with no alterations in their virulence occur in other bacterial species, but have been overlooked in previous studies.

No clear correlation was observed between the virulence and the different phenotypes, except for PF4-R8, which was fully avirulent and had a marked decrease in growth (Figure 1A), motility (Figure 2) and a biochemical profile with several differences to PF4 (Figure 1B). On the other hand, the PF4-R7 strain, for example, has impaired growth and a clear alteration in its biochemical and LPS profile; but not in its virulence compared to PF4 (Figure 6). Furthermore, some of the resistant mutants showed an increased activity in virulence factors such as biofilm production (PF4-R1, PF4-R4 and PF4-R6) or a higher proteolytic activity (PF4-R1, PF4-R2, PF4-R3 and PF4-R9). However, according to our results these alterations are not associated with an increase in their virulence as evaluated using gilthead seabream larvae assay (Figure 6). This result shows that an increased activity in virulence factors does not necessarily imply an increase in the virulence of *V. anguillarum*.

### Correlation Between the Genome of Phage-Resistant Mutants and Implications in Phenotype and Virulence

The phage-resistant mutant PF4-R6 accumulated the greatest number of mutations among the sequenced strains (Figure 8, 9). However, it is not the strain with the lowest virulence, suggesting that there is not a correlation between the number of mutations and the reduction in virulence. This mutant harbored mutations in genes that code for two proteins involved in LPS biosynthesis, which correlates with an altered pattern in the LPS profile observed through SDS-PAGE (Figure 4). The mutations were in the genes coding for ADP-heptose-LPS heptosyltransferase II and LPS chain-length determining protein. In *Salmonella enterica* serovar Typhi, the deletion of the gene that codes for the LPS chain-length determining protein results in cells with variable length in the O-PS chain and, consequently, high susceptibility to the bactericidal action of serum (Hoare et al., 2006). In *V. anguillarum* the length of the O-PS chain is a determinant of virulence, since it has been reported that bacteria that have short chains are more susceptible to the action of the complement (Boesen et al., 1999). On the other hand, the protein ADP-heptose-LPS heptosyltransferase II is involved in the transfer of residues of L,D-heptose to the core oligosaccharide (Nesper et al., 2002a,b; Raetz and Whitfield, 2002). In *V. cholerae*, mutants for this gene present alterations in LPS profile observed by SDS-PAGE with a faster migration of the core OS and absence of O-PS. These mutants also showed a marked deficiency in colonization, probably due to their low resistance to the action of bile and polycationic peptides and a significant reduction in their growth (Nesper et al., 2002b). The PF4-R4 strain also has a mutation in the ADP-heptose-LPS heptosyltransferase II, showing a similar LPS profile to the PF4-R6 strain (Figure 4). Interestingly, the PF4-R4 strain did not show alterations in the virulence on gilthead seabream larvae compared to the parental PF4 strain, suggesting that at least these alterations in the LPS are not affecting the virulence of *V. anguillarum* in this infection model (Figure 6).

Additionally, the PF4-R6 strain had mutations in the genes coding for Sigma-54-dependent Fis family transcriptional regulator (VanO) and the TetR/AcrR family transcriptional regulator (VanT) that are associated with quorum sensing (QS). In *V. anguillarum*, the VanT protein, positively regulates the expression of the metalloproteases EmpA and PrtV, the production of pigments, the production of exopolysaccharide, the biosynthesis of serine and biofilm formation and negatively regulates the expression of the Hcp protein, main component of the type VI secretion (T6SS); while VanO represses the expression of VanT (Croxatto et al., 2002; Weber et al., 2011). Tan et al. (2015) showed that *V. anguillarum* biofilm production is negatively regulated by VanT. When this protein is produced, it reduces biofilm production. According to our results, the PF4-R6 strain shows an increase in biofilm production (Figure 5). This could be explained by the mutations in VanO and VanT in this strain. However, additional analyses are needed to determine how these mutations result in the observed phenotype. The PF4-R4 strain also presented a high production of biofilm; however, it was not possible to establish a relation between the mutations observed in this strain and phenotype, since no mutations were detected in genes that code for proteins involved in the regulation of biofilm production in this mutant. Our analysis was focused on mutations placed in coding regions, therefore one possibility is that this strain has mutations in regulatory regions that alter expression of specific genes associated to biofilm production.

Another interesting mutation was observed in the PF4-R8 strain, which was totally avirulent. This mutant had a deletion of 67 bp in the *rpoN* gene that codes for the RNA polymerase sigma factor 54 (RpoN). In *V. anguillarum* this protein is responsible for the expression of flagellin subunits and mutants in this gene are aflagellated, non-motile and avirulent when fish are infected by immersion (O’Toole et al., 1997). Therefore, this mutation perfectly explains the non-motile and avirulent phenotype observed in the mutant PF4-R8. Moreover, transmission electron microscopy revealed that this mutant did not have flagellum (see Supplementary Figure S4). Nevertheless, the gene *rpoN* participates in the regulation of several other functions besides motility in the bacteria such as biofilm formation and exopolysaccharide production (Hao et al., 2013). Therefore, it cannot be ruled out that other impaired virulence functions are contributing to the avirulent phenotype of the mutant PF4-R8.

The three mutant strains PF4-R4, PF4-R6 and PF4-R8 harbor mutations in the gene coding for the glucose-6-phosphate dehydrogenase protein (NP_233281.1). This gene is essential for bacterial metabolism, however, in the *V. anguillarum* PF4 strain the gene is duplicated (data not shown), explaining why these mutants are viable despite this mutation.

### Prophage Excision in Mutant PF4-R6 of *V. anguillarum*

The resistant mutant PF4-R6 had two large deletions in both chromosomes I and II, corresponding to a prophage that was integrated twice in the bacterial genome (Figure 9). This bacteriophage is very similar to the filamentous phage VCYϕ, of high prevalence in environmental isolates of *Vibrio cholerae* (Xue et al., 2012). Both, the prophage present in *V. anguillarum* strains and the phage VCYφ carry a gene that encodes the Zot toxin, whose function is to increase intestinal permeability by interacting with specific receptors in cells, activating then a series of intracellular signals that cause dismantling of intercellular tight junctions (Di Pierro et al., 2001). The presence of prophages carrying Zot-like proteins have been previously described in the *Vibrio* genus such as *V. coralliilyticus* (Weynberg et al., 2015), *V. anguillarum* strains T265 and Ba35 (Castillo et al., 2017), *V. parahaemolyticus* (Castillo et al., 2018b) and more than 60 other *Vibrio* species (Castillo et al., 2018a). Moreover, recently Castillo et al. (2019) showed that *V. anguillarum* PF4 strains resistant to the broad host range phage KVP40 (Tan et al., 2015) also lost the Zot-encoded prophage. This suggests a possible common outcome independent of which phage is used as selective pressure.

### Final Considerations

Our results show that the phage-resistant mutants of *V. anguillarum* display a greater variability in their phenotype compared to natural variants that occur spontaneously without the pressure of phage infection (see Supplementary Figure S3 and Supplementary Tables S3–S5). The most likely explanation for this phenomenon is that resistance to CHOED is acquired through spontaneous mutation and that the phage simply acts as a selective pressure that selects for resistant mutants, which may have several other alterations besides bacteriophage resistance. In the absence of CHOED, these resistant mutants would be displaced by the wild-type strains of *V. anguillarum* due to their impairment in several features (e.g., growth). However, in the presence of CHOED, the wild-type cells of *V. anguillarum* will be lysed by the phage, allowing the growth of the resistant mutants with multiple phenotypes. As such, the presence of lytic bacteriophages is a driver of diversification and evolution of the bacteria, selecting bacteria with varied phenotypes, which may even have the ability to metabolize new compounds, as our results suggest (Figure 1B). In fact, a study of *Pseudomonas fluorescens* showed that co-evolution with bacteriophages can increase mutation frequency in the bacteria, thus favoring evolution (Pal et al., 2007).

The experiments with gilthead seabream larvae showed that the majority of the bacteriophage resistant mutants had a reduction in their virulence; however, this is not a universal phenomenon, since two phage-resistant mutants remained as virulent as the parental strain. In this regard, the evidence presented in this study is another issue to be considered in the use of bacteriophages as antimicrobials, particularly when they are released into the environment (Meaden and Koskella, 2013). Though several alternatives have been proposed to deal with the problem of proliferation of bacteriophage-resistant strains (Chan and Abedon, 2012; Gu et al., 2012; Pires et al., 2016 and Yen et al., 2017), it should not be taken lightly and these results underline the need for in-depth study of the interactions between bacteriophages and their host bacteria before using them actively as antimicrobial agents.

## Data Availability

The datasets generated for this study can be found in NCBI GenBank, CP023291, CP023290, CP023289, CP023288, CP023433, CP023432, CP023293, and CP023292.

## Author Contributions

ML, PK, DC, KG, and RB conceived and designed the study and analyzed the results. ML performed the experiments. ML, CK, and PK performed the experiments with gilthead seabream larvae (*S. aurata*). ML and DC performed the computational analyses. ML PK, DC, KG, and RB wrote the manuscript. All authors reviewed and approved the final manuscript.

## Conflict of Interest Statement

The authors declare that the research was conducted in the absence of any commercial or financial relationships that could be construed as a potential conflict of interest.

## Footnotes

1 http://www.quantpsy.org/chisq/chisq.htm
