# Supplementary material for: Diversification of Vibrio anguillarum Driven by the Bacteriophage CHOED
Source: Front Microbiol. 2019 Jun 20;10:1396. doi: 10.3389/fmicb.2019.01396 (PMC6596326; doi:10.3389/fmicb.2019.01396)
Supplement: Supplementary file 1 [file Data_Sheet_1.docx]

***Supplementary Material***

**Supplementary figures**


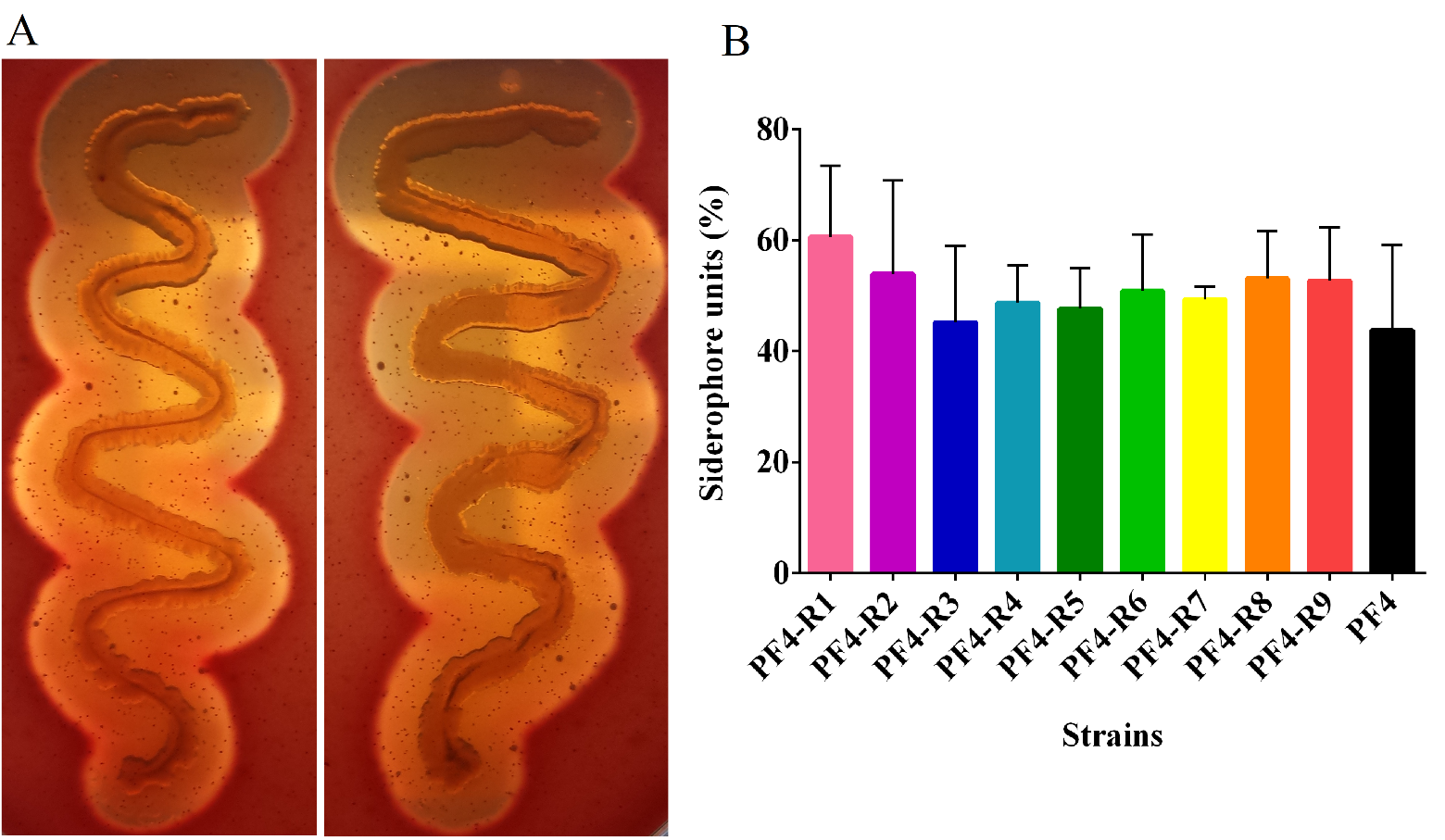


**Figure S1. Haemolytic activity and siderophore production.** A) The image show haemolytic activity in fish-blood agar plates for PF4-R8 (Left) and PF4 *V. anguillarum* (right). B) Siderophore production of the strains resistant to the phage CHOED and the parent strain of *V. anguillarum*. Error bars represent standard deviation of triplicates.


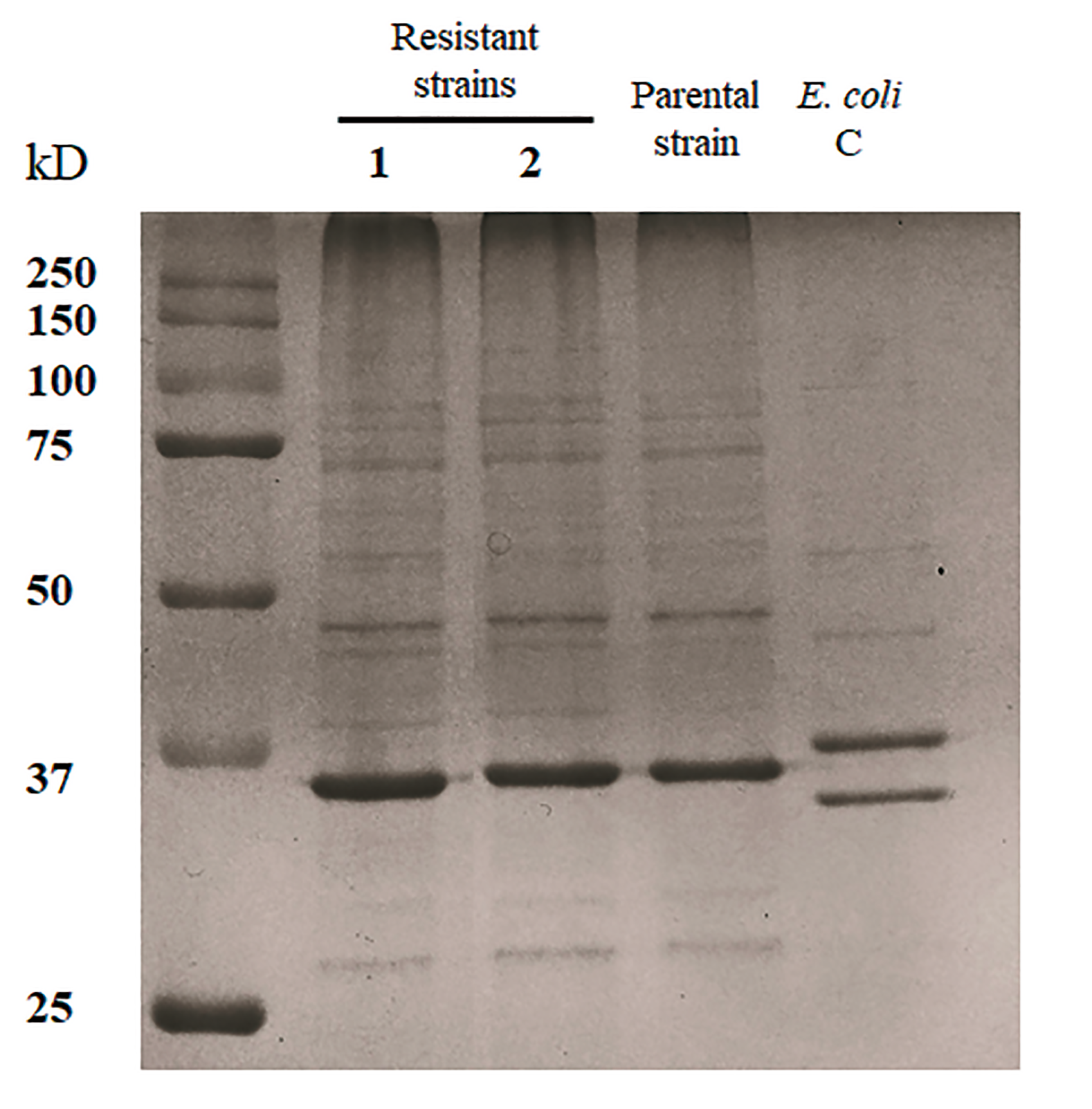


**Figure S2. OMP Profiles.** OMP profiles of some representative strains resistant to the phage CHOED. 1: PF4-R1, 2: PF4-R4, 3: parent strain PF4 and 4: *Escherichia coli* C. Include the molecular weight marker KoleidoscopeTM (BIORAD) in the first left-hand column and the sizes are shown in the figure.


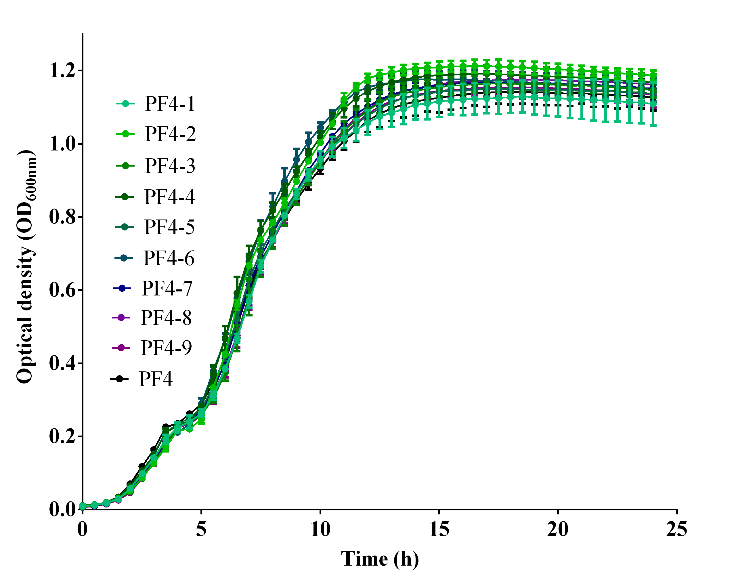


**Figure S3. Growth curve of natural variants of *V. anguillarum*. PF4 is the *wt* parent strain.** The error bars indicate standard deviation. The cultures were performed in quadruplicate.


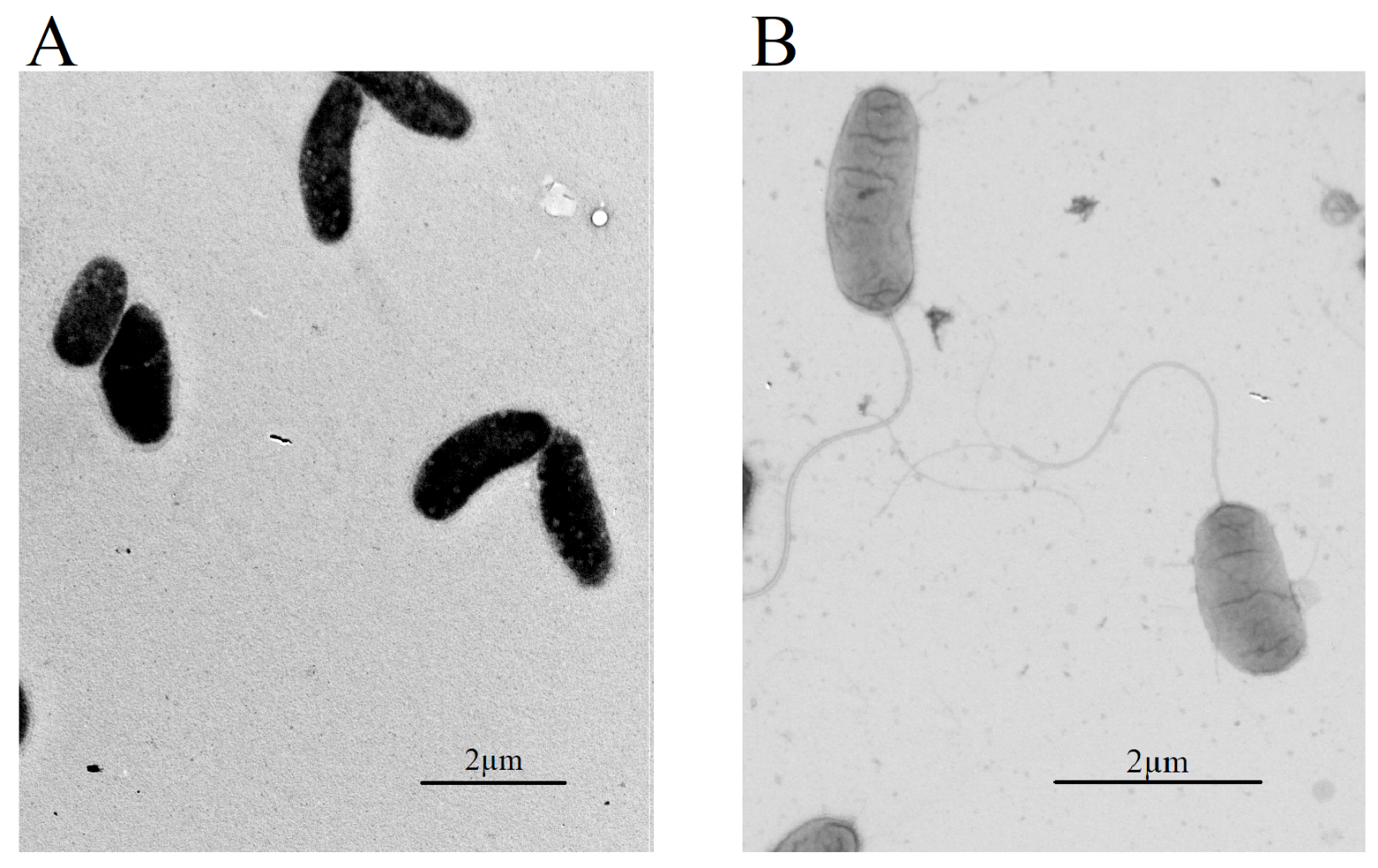


**Figure S4. Transmission electron microscopy.** A) Mutant resistant PF4-R8. B) Parental strain PF4.

**Supplemental tables**

**Tabla S1:** results of Chi-squared analysis (χ2) of the data obtained from the challenges of fish larvae with the resistant strains using the cumulative mortalities at the end of the experiment. The white color indicates that the differences are significant and the blue color indicates that they are not. *P* values of <0.05 were considered statistically signiﬁcant.

| Mutant strains | PF4-R1 | | PF4-R2 | | PF4-R3 | | PF4-R4 | | PF4-R5 | | PF4-R6 | | PF4-R7 | | PF4-R8 | | PF4-R9 | | PF4 | | Control | |
| --- | --- | --- | --- | --- | --- | --- | --- | --- | --- | --- | --- | --- | --- | --- | --- | --- | --- | --- | --- | --- | --- | --- |
|  | chi2 | p | chi2 | p | chi2 | p | chi2 | p | chi2 | p | chi2 | p | chi2 | p | chi2 | p | chi2 | p | chi2 | p | chi2 | p |
| PF4-R1 | ̶̶̶ | **̶** | 31.156 | 0 | 84.103 | 0 | 5.404 | 0,020 | 7.262 | 0,007 | 61.116 | 0 | 13.369 | 0,0003 | 136.878 | 0 | 0 | 1 | 5.404 | 0,020 | 153.783 | 0 |
| PF4-R2 | 31.156 | 0 | **̶** | **̶** | 14.903 | 0 | 59.402 | 0 | 8.738 | 0,003 | 5.657 | 0,0174 | 78.342 | 0 | 45.487 | 0 | 31.156 | 0 | 59.402 | 0 | 55.649 | 0 |
| PF4-R3 | 84.103 | 0 | 14.903 | 0 | **̶** | **̶** | 123.603 | 0 | 44.967 | 0 | 2.272 | 0,1317 | 147.477 | 0 | 9.485 | 0,002 | 84.103 | 0 | 123.603 | 0 | 15.084 | 0 |
| PF4-R4 | 5.404 | 0,020 | 59.402 | 0 | 123.603 | 0 | **̶** | **̶** | 24.425 | 0 | 96.731 | 0 | 1.914 | 0,1665 | 182.247 | 0 | 5.404 | 0,020 | 0 | 1 | 200.402 | 0 |
| PF4-R5 | 7.262 | 0,007 | 8.738 | 0,003 | 4.4967 | 0 | 24.425 | 0 | **̶** | **̶** | 27.902 | 0 | 38.252 | 0 | 88.469 | 0 | 7.262 | 0,007 | 24.425 | 0 | 200.402 | 0 |
| PF4-R6 | 61.116 | 0 | 5.657 | 0,017 | 2.272 | 0,132 | 96.731 | 0 | 27.902 | 0 | **̶** | **̶** | 118.927 | 0 | 20.467 | 0 | 61.116 | 0 | 96.731 | 0 | 103.346 | 0 |
| PF4-R7 | 13.369 | 0 | 78.342 | 0 | 147.477 | 0 | 1.914 | 0,167 | 38.252 | 0 | 118.927 | 0 | **̶** | **̶** | 208.602 | 0 | 13.369 | 0 | 1.914 | 0,167 | 29.422 | 0 |
| PF4-R8 | 136.878 | 0 | 45.487 | 0 | 9.485 | 0,002 | 182.247 | 0 | 88.469 | 0 | 20.467 | 0 | 208.602 | 0 | **̶** | **̶** | 136.878 | 0 | 182.247 | 0 | 2.339 | 0,126 |
| PF4-R9 | 0 | 1 | 31.156 | 0 | 84.103 | 0 | 5.404 | 0,020 | 7.262 | 0,007 | 61.116 | 0 | 13.369 | 0,0003 | 136.878 | 0 | **̶** | **̶** | 5.404 | 0,020 | 874.082 | 0 |
| PF4 | 5.404 | 0,020 | 59.402 | 0 | 123.603 | 0 | 0 | 1 | 24.425 | 0 | 96.731 | 0 | 1.914 | 0,1665 | 182.247 | 0 | 5.404 | 0,020 | ̶ | **̶** | 153.783 | 0 |
| Control | 153.783 | 0 | 55.649 | 0 | 15.084 | 0 | 200.402 | 0 | 200.402 | 0 | 103.346 | 0 | 29.422 | 0 | 2.339 | 0,126 | 874.082 | 0 | 200.402 | 0 | **̶** | **̶** |

**Tabla S2: results of Chi-squared analysis (χ2) of the data obtained from the challenges of fish larvae with the natural variants using the cumulative mortalities at the end of the experiment. The white color indicates that the differences are significant and the blue color indicates that they are not. *P* values of <0.05 were considered statistically signiﬁcant.**

| Natural variants | PF4-1 | | PF4-2 | | PF4-3 | | PF4-4 | | PF4-5 | | PF4-6 | | PF4-7 | | PF4-8 | | PF4-9 | | PF4 | | Control | |
| --- | --- | --- | --- | --- | --- | --- | --- | --- | --- | --- | --- | --- | --- | --- | --- | --- | --- | --- | --- | --- | --- | --- |
|  | chi2 | p | chi2 | p | chi2 | p | chi2 | p | chi2 | p | chi2 | p | chi2 | p | chi2 | p | chi2 | p | chi2 | p | chi2 | p |
| PF4-1 | ̶̶̶ | **̶** | 1.243 | 0,265 | 1.852 | 0,174 | 0,279 | 0,597 | 11.096 | 0 | 4.758 | 0,029 | 0,92 | 0,338 | 6.692 | 0,010 | 5.722 | 0,017 | 1.909 | 0,167 | 161.049 | 0 |
| PF4-2 | 1.243 | 0,265 | ̶ | ̶ | 0,061 | 0,805 | 0,338 | 0,561 | 5.002 | 0,025 | 1,14 | 0,286 | 0,024 | 0,877 | 2.205 | 0,138 | 1.669 | 0,196 | 6.164 | 0,013 | 14.656 | 0 |
| PF4-3 | 1.852 | 0,174 | 0,061 | 0,805 | ̶ | ̶ | 0,683 | 0,409 | 3.975 | 0,046 | 0,674 | 0,412 | 0,16 | 0,689 | 1,54 | 0,215 | 1.099 | 0,295 | 7.429 | 0,006 | 192.392 | 0 |
| PF4-4 | 0,279 | 0,597 | 0,338 | 0,561 | 0,683 | 0,409 | ̶ | ̶ | 7.835 | 0,005 | 2.702 | 0,100 | 0,183 | 0,669 | 4.218 | 0,040 | 3.462 | 0,063 | 3.592 | 0,058 | 20.039 | 0 |
| PF4-5 | 11.096 | 0 | 5.002 | 0,025 | 3,975 | 0,046 | 7.835 | 0,005 | ̶ | ̶ | 1.406 | 0,236 | 5.688 | 0,017 | 0,569 | 0,451 | 0,451 | 0,346 | 21.648 | 0 | 239.352 | 0 |
| PF4-6 | 4.758 | 0,029 | 1,14 | 0,286 | 0,674 | 0,412 | 2.702 | 0,100 | 1.406 | 0,236 | ̶ | ̶ | 1.488 | 0,223 | 0,183 | 0,669 | 0,056 | 0,813 | 12.511 | 0 | 212.902 | 0 |
| PF4-7 | 0,92 | 0,338 | 0,024 | 0,877 | 0,16 | 0,689 | 0,183 | 0,669 | 5.688 | 0,017 | 1.488 | 0,223 | ̶ | ̶ | 2.674 | 0,102 | 2,08 | 0,149 | 5.415 | 0,020 | 182.425 | 0 |
| PF4-8 | 6.692 | 0,009 | 2.205 | 0,138 | 1,54 | 0,215 | 4.218 | 0,040 | 0,569 | 0,451 | 0,183 | 0,669 | 2.674 | 0,102 | ̶ | ̶ | 0,036 | 0,036 | 15.395 | 0 | 220.703 | 0 |
| PF4-9 | 5.722 | 0,017 | 1.669 | 0,196 | 1.099 | 0,295 | 3.462 | 0,063 | 0,451 | 0,346 | 0,056 | 0,813 | 2,08 | 0,149 | 0,036 | 0,036 | ̶ | ̶ | 13,9 | 0 | 215.118 | 0 |
| PF4 | 1.909 | 0,167 | 6.164 | 0,013 | 7.429 | 0,006 | 3.592 | 0,058 | 21.648 | 0 | 12.511 | 0 | 5.415 | 0,020 | 15.395 | 0 | 13,9 | 0 | ̶ | **̶** | 129.125 | 0 |
| Control | 161.049 | **0** | 14.656 | 0 | 192.392 | 0 | 20.039 | 0 | 239.352 | 0 | 212.902 | 0 | 182.425 | 0 | 220.703 | 0 | 215.118 | 0 | 129.125 | **0** | **̶** | **̶** |

**Table S3:** Calculation of the coefficient of variation (CV) of the results of the % cumulative mortality of challenged larvae with phage resistant strains and natural variants of *V. anguillarum* PF4, at the end of the experiment (7 days).

| Natural variants | % Cumulative mortality of natural variants | Resistant strains | % Cumulative mortality of phage resistant strains |
| --- | --- | --- | --- |
| PF4-1 | 68,9 | PF4-R1 | 73,9 |
| PF4-2 | 74,3 | PF4-R2 | 45,0 |
| PF4-3 | 75,4 | PF4-R3 | 25,6 |
| PF4-4 | 71,5 | PF4-R4 | 83,9 |
| PF4-5 | 84,0 | PF4-R5 | 60,6 |
| PF4-6 | 79,1 | PF4-R6 | 32,8 |
| PF4-7 | 73,7 | PF4-R7 | 88,9 |
| PF4-8 | 80,9 | PF4-R8 | 12,8 |
| PF4-9 | 80,1 | PF4-R9 | 73,9 |
| PF4 | 61,9 | PF4 | 83,9 |
| **Average** | 75,0 | **Average** | 58,1 |
| **Standard deviation (SD)** | 6,5 | **Standard deviation (SD)** | 27,3 |
| **Coefficient of variation (CV)** | 8,7 | **Coefficient of variation (CV)** | 47 |

**Table S4:** Calculation of the coefficient of variation (CV) of the biofilm production results of phage resistant strains and natural variants of *V. anguillarum* PF4.

| Natural variants | Biofilm production of natutal variants (O.D._550nm_) | Resistant strains | Biofilm production of phage resistant strains (O.D._550nm_) |
| --- | --- | --- | --- |
| PF4-1 | 0,52 | PF4-R1 | 0,47 |
| PF4-2 | 0,61 | PF4-R2 | 0,21 |
| PF4-3 | 0,53 | PF4-R3 | 0,26 |
| PF4-4 | 0,63 | PF4-R4 | 1,21 |
| PF4-5 | 0,55 | PF4-R5 | 0,17 |
| PF4-6 | 0,57 | PF4-R6 | 0,45 |
| PF4-7 | 0,50 | PF4-R7 | 0,13 |
| PF4-8 | 0,52 | PF4-R8 | 0,12 |
| PF4-9 | 0,62 | PF4-R9 | 0,14 |
| PF4 | 0,50 | PF4 | 0,11 |
| **Average** | 0,55 | **Average** | 0,33 |
| **Standard deviation (SD)** | 0,05 | **Standard deviation (SD)** | 0,34 |
| **Coefficient of variation (CV)** | 9,00 | **Coefficient of variation (CV)** | 102,88 |

**Table S5:** Calculation of the coefficient of variation (CV) of the motility results of phage resistant strains and natural variants of *V. anguillarum* PF4.

| Natural variants | Motility (mm) of natural variants | Resistant strains | Motility (mm) of phage resistant strains |
| --- | --- | --- | --- |
| PF4-1 | 52,17 | PF4-R1 | 34,33 |
| PF4-2 | 45,00 | PF4-R2 | 33,33 |
| PF4-3 | 47,67 | PF4-R3 | 35,33 |
| PF4-4 | 58,83 | PF4-R4 | 31,33 |
| PF4-5 | 53,83 | PF4-R5 | 33,67 |
| PF4-6 | 47,67 | PF4-R6 | 39,00 |
| PF4-7 | 49,67 | PF4-R7 | 39,17 |
| PF4-8 | 57,83 | PF4-R8 | 3,67 |
| PF4-9 | 56,17 | PF4-R9 | 39,67 |
| PF4 | 57,33 | PF4 | 35,17 |
| **Average** | 52,62 | **Average** | 32,47 |
| **Standard deviation (SD)** | 4,92 | **Standard deviation (SD)** | 10,50 |
| **Coefficient of variation (CV)** | 9,36 | **Coefficient of variation (CV)** | 32,33 |

**Table S6:** Genes with mutations in *V. anguillarum* phage-resistant strains

| Chromosome I | | | | | | | | | |
| --- | --- | --- | --- | --- | --- | --- | --- | --- | --- |
| Protein accession | ^a^Position (start/stop) | ^a^Position mutation | Type of mutation | bp | Phage-resistant isolates | Function | ^b^Cellular locatization | ^c^TMH/SP | ^d^Effect mutation |
| NP_232157.1 | 227.053-228.513 | 227.780-227.846 | Deletion | 67 | PF4-R8 | RNA polymerase factor sigma-54 (RpoN) | Cy | - / - | ^e^FS |
| WP_017048278.1 | 470.681-469.554 | 469.670-469.676 | Deletion | 7 | PF4-R6 | LPS chain-length determining protein | CyM | + / - | ^e^FS |
| WP_019281202.1 | 489.203-488.169 | 488.227-488.337 | Deletion | 11 | PF4-R4 | ADP-heptose-LPS heptosyltransferase II | Uk | - / - | ^e^FS |
|  |  | 488.653 | Point | 1 | PF4-R6 |  |  |  | SC |
| WP_017049544.1 | 1.596.421-1.596.221 | 1.596.247 | Point | 1 | PF4-R6 | Hypothetical protein | CyM | + / - | AC |
|  |  | 1.596.249 | Point | 1 |  |  |  |  | AC |
|  |  | 1.596.274-1.596.313 | Deletion | 39 |  |  |  |  | ^e^FS |
| WP_013856939.1 | 1.649.388-1.645.114 | 1.649.268 | Insertion | 1 | PF4-R8 | hcalcium-binding protein, RTX toxin-related (FrpC) | ExC | - / - | FS |
| WP_010320686.1 | 1.990.686-1.989.280 | 1.990.115 | Point | 1 | PF4-R6 | Sigma-54-dependent Fis family transcriptional regulator (VanO) | Cy | - / - | AC |
|  |  | 1.990.596 | Point | 1 |  |  |  |  | AC |
| WP_017049502.1 | 2.037.953-2.034.843 | 2.034.848-2.034.843 | Deletion | 6 | PF4-R4 | Hypothetical protein of outer membrane | OM | +/- | FS |
|  |  | 2.034.705 | Insertion | 3 | PF4-R4 |  |  |  | AI |
|  |  | 2.034.835-2.034.843 | Deletion | 9 | PF4-R6 |  |  |  | FS |
|  |  | 2.034.705 | Insertion | 3 | PF4-R6 |  |  |  | AI |
|  |  | 2.034.828-2.034.854 | Deletion | 27 | PF4-R8 |  |  |  | FS |
| WP_013857308.1 | 2.054.712-2.055.578 | 2.054.904-2.054.929 | Deletion | 26 | PF4-R8 | Transcriptional regulator (ToxR) | Uk | + / - | ^e^FS |
| NP_797001.1 | 2.219.594-2.221.138 | 2.219.817 | Point | 1 | PF4-R8 | Alanine:cation symporter family protein | CyM | + / - | AC |
|  |  | 2.219.829 | Point | 1 | PF4-R4 |  |  |  | AC |
| WP_010319157.1 | 2.300.321-2.302.000 | 2.301.651 | Point | 1 | PF4-R6 | Sensor histidine kinase (YehU) | CyM | + / - | AC |
| NP_798862.1 | 2.392.377-2.393.369 | 2.392.498 | Insertion | 8 | PF4-R6 | ABC transporter ATP-binding protein | CyM | - / - | AI |
|  |  | 2.392.503-2.392.504 | Deletion | 2 |  |  |  |  |  |
|  |  | 2.392.510 | Insertion | 1 |  |  |  |  | FS |
|  |  | 2.392.536 | Point | 1 |  |  |  |  | SC |
|  |  | 2.392.538 | Point | 1 |  |  |  |  |  |
| WP_017043694.1 | 2.433.927-2.434.544 | 2.434.475 | Insertion | 5 | FP4-R6 | TetR/AcrR family transcriptional regulator (VanT) | Cy | - / - | ^e^FS |
| WP_017046665.1 | 2.593.145-2.595.718 | 2.593.754 | Insertion | 1 | PF4-R8 | Protein of unknown function | CyM | + / - | ^e^FS |
| WP_005521764.1 | 2.825.992-2.825.714 | 2.825.987-2.834.894 | Deletion | 8.905 | PF4-R6 | Glyoxalase | Uk | - / - | FS |
| WP_006880472.1 | 2.826.045-2.826.413 | 2.825.987-2.834.894 | Deletion | 8.905 | PF4-R6 | Transcription Regulator | Cy | - / - | D |
|  | 2.827.391-2.826.453 | 2.825.987-2.834.894 | Deletion | 8.905 | PF4-R6 | Hypothetical protein | CyM | + / - | D |
|  | 2.827.783-2.827.400 | 2.825.987-2.834.894 | Deletion | 8.905 | PF4-R6 | Hypothetical protein | CyM | + / - | D |
| WP_017041958.1 | 2.829.680-2.828.316 | 2.825.987-2.834.894 | Deletion | 8.905 | PF4-R6 | Zonula occludens toxin (Zot) | Uk | + / - | D |
| WP_019281837.1 | 2.830.027-2.829.683 | 2.825.987-2.834.894 | Deletion | 8.905 | PF4-R6 | Protein of unknown function | CyM | + / - | D |
| WP_017049549.1 | 2.831.349-2.830.027 | 2.825.987-2.834.894 | Deletion | 8.905 | PF4-R6 | Minor capsid protein | CyM | + / - | D |
| WP_017041960.1 | 2.831.702-2.831.484 | 2.825.987-2.834.894 | Deletion | 8.905 | PF4-R6 | Hypothetical protein | Uk | + /+ | D |
| WP_017049548.1 | 2.831.926-2.831.732 | 2.825.987-2.834.894 | Deletion | 8.905 | PF4-R6 | Hypothetical protein | Uk | - / - | D |
| WP_017049547.1 | 2.832.327-2.831.962 | 2.825.987-2.834.894 | Deletion | 8.905 | PF4-R6 | Hypothetical protein | Cy | - / - | D |
| WP_017049546.1 | 2.833.524-2.832.334 | 2.825.987-2.834.894 | Deletion | 8.905 | PF4-R6 | DNA replication initiation protein | Cy | - / - | D |
| WP_017049545.1 | 2.833.814-2.833.617 | 2.825.987-2.834.894 | Deletion | 8.905 | PF4-R6 | Hypothetical protein | Uk | - / - | D |
| WP_009706113.1 | 2.834.383-2.833.817 | 2.825.987-2.834.894 | Deletion | 8.905 | PF4-R6 | 3'-5' exonuclease | Uk | - / - | D |
| WP_019276426.1 | 2.834.551-2.834.922 | 2.825.987-2.834.894 | Deletion | 8.905 | PF4-R6 | Hypothetical protein | Uk | - / - | D |
| WP_017045972.1 | 2.845.211-2.845.522 | 2.845.201 | Insertion | 1 | PF4-R8 | 1,4-alpha-glucan-branching protein | Uk | - / - | FS |
| Chromosome II | | | | | | | | | |
| nº accession | ^a^Position (start/stop) | ^a^Position mutation | Type of mutation | Pb | Phage-resistant isolates | Function | ^b^Cellular locatization | ^c^TMH/SP | ^d^Effect mutation |
| NP_230280.1 | 1-570 | 564-565 | Deletion | 2 | All | Tyrosine tRNA ligase | Cy | - / - | FS |
| WP_017048771.1 | 131.254-129.902 | 131.077 | Point | 1 | PF4-R6 | ABC transporter substrate-binding protein | Uk | - / - | AC |
| WP_000931488.1 | 131.595-132.914 | 131.539 | Insertion | 1 | PF4-R6 | MFS transporter | CyM | + / - | FS |
| NP_233281.1 | 439.947-440.915 | 440.053 | Insertion | 2 | All | glucose-6-phosphate dehydrogenase | Cy | - / - | ^e^FS |
| WP_017044929.1 | 903.989-904.813 | 904.182 | Point | 1 | PF4-R8 | AraC family transcriptional regulator | Cy | - / - | AC |
| WP_001899004.1 | 940.057-940.253 | 940.207 | Deletion | 1 | PF4-R4 | hypothetical protein | Cy | - / - | ^e^FS |
| WP_019820677.1 | 998.201-997.944 | 998.189-1.007.771 | Deletion | 9582 | PF4-R6 | Glyoxalase | Uk | - / - | FS |
| WP_006880472.1 | 998.268-998.636 | 998.245-1.011.237 | Deletion | 9582 | PF4-R6 | Transcription Regulator | Cy | - / - | D |
|  | 999.614-998.676 | 998.245-1.011.237 | Deletion | 9582 | PF4-R6 | Hypothetical protein | CyM | + / - | D |
|  | 1.000.006-999.623 | 998.245-1.011.237 | Deletion | 9582 | PF4-R6 | Hypothetical protein | CyM | + / - | D |
| WP_017041958.1 | 1.001.903-1.000.539 | 998.245-1.011.237 | Deletion | 9582 | PF4-R6 | Zonula occludens toxin (Zot) | Uk | + / - | D |
| WP_019281837.1 | 1.002.250-1.001.906 | 998.245-1.011.237 | Deletion | 9582 | PF4-R6 | Protein of unknown function | CyM | + / - | D |
| WP_017049549.1 | 1.003.572-1.002.250 | 998.245-1.011.237 | Deletion | 9582 | PF4-R6 | minor capsid protein | CyM | + / - | D |
| WP_017041960.1 | 1.003.925-1.003.707 | 998.245-1.011.237 | Deletion | 9582 | PF4-R6 | Hypothetical protein | Uk | + / + | D |
| WP_017049548.1 | 1.004.149-1.003.955 | 998.245-1.011.237 | Deletion | 9582 | PF4-R6 | Hypothetical protein | Uk | - / - | D |
| WP_017049547.1 | 1.004.550-1.004.185 | 998.245-1.011.237 | Deletion | 9582 | PF4-R6 | Hypothetical protein | Cy | - / - | D |
| WP_017049546.1 | 1.005.747-1.004.557 | 998.245-1.011.237 | Deletion | 9582 | PF4-R6 | DNA replication initiation protein | Cy | - / - | D |
| WP_017049545.1 | 1.006.037-1.005.840 | 998.245-1.011.237 | Deletion | 9582 | PF4-R6 | Hypothetical protein | Uk | - / - | D |
| WP_009706113.1 | 1.006.606-1.006.040 | 998.245-1.011.237 | Deletion | 9582 | PF4-R6 | 3'-5' exonuclease | Uk | - / - | D |
| WP_017049544.1 | 1.006.774-1.007.142 | 998.245-1.011.237 | Deletion | 9582 | PF4-R6 | Hypothetical protein | Uk | - / - | D |

^a^Position (start/stop)/ ^a^Position mutation: Position of Protein or mutation in *Vibrio anguillarum* PF4

^b^Cellular localization: Cytoplasmic (Cy); Cytoplasmic membrane (CyM); Outer membrane (OM); Extracellular (ExC); Unknown (Uk).

^c^THM/SP: Transmembrane helice/Signal peptide.

^d^Effect mutation: amino acid change (AC); amino acid insertion (AI); frameshift (FS); stop codon (SC); Deletion full gene (D).

^e^FS: mutation caused frameshift but the gene also presented a stop codon.

**Table S7**. List of prophages found in the genome of *V. anguillarum* PF4.

| Prophage | Size (kb) | #ORFs | %CG | Position^a^ (chromosome) | Status | Most Common Phage |
| --- | --- | --- | --- | --- | --- | --- |
| 1 | 9,8 | 16 | 40,34 | 2825037-2834913 (CI) | intact | Phage Vibrio VCYϕ |
|  |  |  | 40,25 | 997270-1007143  (CII) |  |  |
| 2 | 52.7 | 60 | 44,87 | 451331-504074 (CII) | intact | Phage Vibrio VP882 |

^a^position according to PF4 strain

**Table S8**. List of accession numbers for chromosomes the genomic sequences of *Vibrio anguillarum* PF4 strains.

| *Vibrio anguillarum* strain | Chromosome | Accession number |
| --- | --- | --- |
| PF4 | I | CP023291 |
|  | II | CP023290 |
| PF4-R4 | I | CP023289 |
|  | II | CP023288 |
| PF4-R6 | I | CP023433 |
|  | II | CP023432 |
| PF4-R8 | I | CP023293 |
|  | II | CP023292 |
